# Temperature Influences the Production and Transport of Saxitoxin and the Expression of *sxt* Genes in the Cyanobacterium *Aphanizomenon gracile*

**DOI:** 10.3390/toxins9100322

**Published:** 2017-10-13

**Authors:** Samuel Cirés, Adrián Delgado, Miguel González-Pleiter, Antonio Quesada

**Affiliations:** Departamento de Biología, Darwin, 2, Universidad Autónoma de Madrid, 28049 Madrid, Spain; adrian.delgadoo@estudiante.uam.es (A.D.); mig.gonzalez@uam.es (M.G.-P.); antonio.quesada@uam.es (A.Q.)

**Keywords:** cyanobacteria, saxitoxin, transport, extracellular, *sxtA*, *sxtM*, *sxtPer*, NorM-MATE, Drug Metabolite Transporter (DMT), qPCR

## Abstract

The cyanobacterium *Aphanizomenon gracile* is the most widely distributed producer of the potent neurotoxin saxitoxin in freshwaters. In this work, total and extracellular saxitoxin and the transcriptional response of three genes linked to saxitoxin biosynthesis (*sxtA*) and transport (*sxtM*, *sxtPer*) were assessed in *Aphanizomenon gracile* UAM529 cultures under temperatures covering its annual cycle (12 °C, 23 °C, and 30 °C). Temperature influenced saxitoxin production being maximum at high temperatures (30 °C) above the growth optimum (23 °C), concurring with a 4.3-fold increased *sxtA* expression at 30 °C. Extracellular saxitoxin transport was temperature-dependent, with maxima at extremes of temperature (12 °C with 16.9% extracellular saxitoxin; and especially 30 °C with 53.8%) outside the growth optimum (23 °C), coinciding with a clear upregulation of *sxtM* at both 12 °C and 30 °C (3.8–4.1 fold respectively), and yet with just a slight upregulation of *sxtPer* at 30 °C (2.1-fold). Nitrate depletion also induced a high extracellular saxitoxin release (51.2%), although without variations of *sxtM* and *sxtPer* transcription, and showing evidence of membrane damage. This is the first study analysing the transcriptional response of *sxtPer* under environmental gradients, as well as the effect of temperature on putative saxitoxin transporters (*sxtM* and *sxtPer*) in cyanobacteria in general.

## 1. Introduction

Saxitoxins (STXs), also known as Paralytic Shellfish toxins, comprise a group of neurotoxic carbamate alkaloids including the main chemical variant saxitoxin (STX) (C_10_H_17_N_7_O_4_) and up to 57 analogues [1]. STXs are potent blockers of voltage-gated sodium channels present in neuronal cell membranes causing death by respiratory failure, being also able to block calcium channels and to prolong the gating of potassium channels in heart muscle cells [2]. Indeed, STXs include some of the most potent natural toxins known so far with lethal doses in mice (LD_50_) ranging from 10 to30 µg kg^−1^ [3]. Given their high toxicity and demonstrated bioaccumulation in the trophic chain [4,5,6,7], STXs pose a matter of concern for ecosystems and human health around the globe.

STXs are produced by marine (eukaryotic) dinoflagellates, while their production in freshwaters is ascribed only to cyanobacteria (prokaryotes) [8]. Freshwater STXs represent a worldwide phenomenon, as detected in Asia, North and South America, Europe, and Oceania (see [8] for a review), with a growing number of STX reports in the last 10 years including locations like the Arctic [9] or New Zealand [10]. So far STX synthesis has been described in cultures of filamentous cyanobacteria from genera *Anabaena*, *Aphanizomenon*, *Cuspidothrix*, *Cylindrospermopsis*, *Lyngbya*, *Raphidiopsis,* and *Scytonema* [8,11].

Among this diversity of cyanobacterial producers, the Nostocalean *Aphanizomenon gracile* arguably displays the broadest geographic distribution according to data available. In fact, STX-producing strains of *Aph. gracile* have been isolated from lakes and reservoirs in latitudes from 40° to 60° North in Asia (China, Japan), North America, and especially in Europe (Spain, Portugal, France, Germany, and Norway) [11,12]. Given the undoubted toxicological and biogeographical importance of *Aph. gracile*, the lack of molecular studies on the environmental regulation of STX synthesis and transport in this cyanobacterium is striking.

STXs biosynthesis by cyanobacteria occurs via the *sxt* gene cluster, encoding for a complex of enzymes catalysing the synthesis of STX (polyketide synthases -PKS- aminotransferases, cyclases, etc.), transporters and regulatory genes. It has to be noted that the function proposed for *sxt*-encoded proteins remains just putative and is based solely on sequence homology [8]. Sxt cluster varies in length (from 25.7 kb to 35 kb), number of genes (26–31), and arrangement of those in the different producing organisms, suggesting an ancient origin followed by a complex history of gene transfers, deletions, and recombination events [13]. In *Aph. gracile* UAM529—a strain that produces mostly STX and minor amounts of other variants like neosaxitoxin and decarbamoylsaxitoxin [12]—the *sxt* cluster spans for 27.3 kb with 27 open reading frames (orfs), comprising 14 core genes common to all STX producers (such as the PKS-encoding gene *sxtA*) involved in the main enzymatic functions (e.g., condensation, cyclisation, and desaturation), and additional tailoring genes (e.g., *sxtC* gene among others), regulatory genes, and auxiliary genes involved in extracellular transport of STXs (e.g., *sxtM* and *sxtPer*) [12,14]. Among proteins for export, SxtM is an efflux pump belonging to the MATE multidrug and toxic compound extrusion superfamily and is present in all cyanobacterial STX-producers so far, while SxtPer is a permease of the drug/metabolite transporter (DMT) superfamily and has been found only in *Anabaena*, *Aphanizomenon,* and *Lyngbya* [14].

STX transport outside the cell is a priority topic since an important proportion of the toxin (7%–35%) is found extracellularly, even during exponential growth of the producers [15,16,17]. Notably, this extracellular STX represents the fraction in direct contact with ecosystem organisms and water users. It is therefore essential to understand the genetic regulation of putative STX transporters—e.g., *sxtM* and *sxtPer* [12]—under different environmental gradients. Previous molecular works found differences in *sxtM* transcription in *Anabaena circinalis*, *Cylindrospermopsis raciborskii,* and *Raphidiopsis brookii* under varying pH, Na^+^ concentrations, and nitrogen sources [18,19]. However, there is no information on *sxtM* regulation in *Aph. gracile* and no study on the environmental regulation of *sxtPer* in any species whatsoever. Furthermore, none of the previous studies in cyanobacteria has focused on the influence of temperature on the expression of STX transporters, an aspect of utmost relevance to understand shifts in dissolved toxins throughout the annual cycle of producers or under the global warming scenario.

The present study aims at investigating the effect of temperature on the production and extracellular release of STX by *Aph. gracile* strain UAM529 isolated from a Spanish reservoir. Along with total STX content and the share of extracellular STX, the expression of three genes involved in STX biosynthesis (*sxtA*) and transport (*sxtM*, *sxtPer*) will be analysed in a temperature range covering the vegetative annual cycle of *Aph. gracile*. This will enable the gaining of insight into the influence of temperature on STX synthesis and transport at the transcriptional level, as well as the discussion of its possible implications for the ecology and toxicology of cyanobacteria in the context of their annual life cycle in temperate freshwaters.

## 2. Results

### 2.1. Ecophysiology of Aphanizomenon Gracile

Over the 8-day experimental period, cultures of *Aph. gracile* UAM529 showed a similar growth pattern at the three temperatures tested (12 °C, 23 °C, and 30 °C) (Figure S1 in Supplementary Materials), with a short lag phase of about two days and exponential growth thereafter until day 8. None of the cultures reached the stationary phase, yet after day 5, cultures at 12 °C and 30 °C initiated the late exponential growth phase. The maximum final biomass after 8 days was reached at 23 °C (0.51 g dry weight L^−1^) followed by 12 °C (0.42 g DW L^−1^), with the lowest biomass reached at 30 °C (0.27 g DW L^−1^) (Figure S1 in Supplementary Materials).

Temperature clearly affected growth rates (µ), with the maximum rate reached at the intermediate temperature of 23 °C (µ = 0.17 day^−1^) significantly higher (*p* < 0.05; one-way ANOVA) than the extremes of temperature of 12 °C (µ = 0.10 day^−1^) and 30 °C (µ = 0.07 day^−1^) (Table 1). The Chl *a* content followed the same temperature-dependent pattern, with the maximum average content reached at 23 °C (13.6 mg Chl *a* g^−1^ DW), significantly higher than Chl *a* contents of 12 °C and 30 °C (8.6 and 9.9 mg Chl *a* g^−1^ DW, respectively) (Table 1).

Beyond growth over time, we analysed the properties of cell membranes after 8 days of growth at the different temperatures by means of two different fluorochromes: Propidium Iodide (PI) for membrane integrity, and DiBAC_4_(3) for membrane potential (Table S1 from Supplementary Materials). Fluorescence values were compared using the temperature with the optimal growth (23 °C) as reference in order to find alterations in membranes at temperatures away from the optimal physiological status. On day 8, cultures from the three temperatures showed no significant differences in membrane integrity, with very similar PI fluorescence (Table S1 from Supplementary Materials). However, we could observe a significant membrane hyperpolarisation at 30 °C compared to 23 °C, evidenced by a sharp decrease in DiBAC_4_(3) fluorescence (Table S1 from Supplementary Materials).

### 2.2. Effects of Temperature on Saxitoxin Production and Release

**Figure 1:**
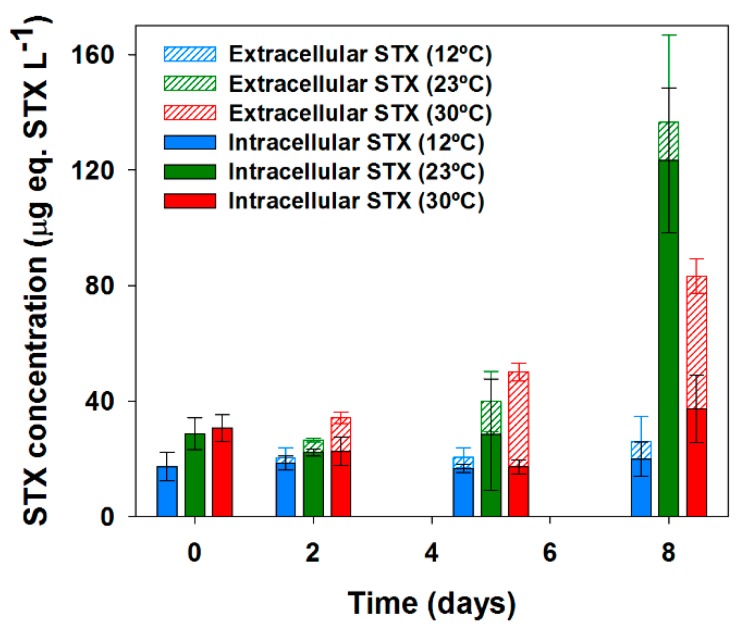
Over-time dynamics of intra and extracellular saxitoxins in *Aphanizomenon gracile* UAM529 under three different temperatures. Error bars indicate standard deviation of three replicates (*n* = 3).

*Aph. gracile* UAM529 produced moderate concentrations of total STX (sum of intracellular and extracellular STX), detectable at the three temperatures varying from 17.3 to 136.5 µg equiv. STX L^−1^ over the entire experimental range (Figure 1). Total saxitoxin concentrations increased over time at the three temperatures (Figure 1), reaching maxima on day 8 in all cases. On day 8, the maximum concentration was detected in the cultures at 23 °C (136.5 µg equiv. STX L^−1^), being 64% higher than that at 30 °C (83.3 µg STX equiv. L^−1^) and with a minimum at 12 °C up to 5-fold lower than that measured at 23 °C.

Temperature clearly influenced not just the total STX concentration (µg STX equiv. L^−1^) but also the STX standardized to biomass (µg equiv. STX mg^−1^ DW), hereafter STX content (Table 2). On average, cultures at 23 °C and 30 °C showed very similar STX contents of 0.20 and 0.25 µg equiv. STX mg^−1^ DW, respectively, while STX content decreased drastically at 12 °C, being 2.4–3.2 fold lower than the contents of the other two temperatures.

Extracellular STX was detectable in all experiments after 2 days of growth, representing a notable share of the total STX (ranging 9%–65% in the different temperatures) that was especially evident after day 5 of growth (Figure 1). Temperature triggered important differences in the share of extracellular STX (Table 2) but with a pattern differing from that observed for the total STX content. In this case, the average extracellular share was maximum at 30 °C (53.8%), being significantly higher (up to 4.5-fold) than those at 12 °C and 23 °C, which showed similar average extracellular STX shares of 11.8% and 16.9%, respectively (Table 2).

### 2.3. Effects of Temperature on the Expression of sxt Genes

#### 2.3.1. Dynamics of STX-Biosynthesis Gene *sxtA*

Transcripts of the gene *sxtA*—encoding a PKS putatively catalysing the first step of STX biosynthesis—were present at the three temperatures studied (12 °C, 23 °C, and 30 °C) throughout the growth period (from day 0 to day 8). This enabled describing the dynamics of expression of *sxtA* related to temperature over time. Relative expression values were calculated using data from 23 °C as control (hence setting its relative expression as 1) as the condition where *Aph. gracile* UAM529 showed the best physiological status of the study—highest growth rate and Chla content and no signs of membrane damages—according to the results shown above.

**Figure 2:**
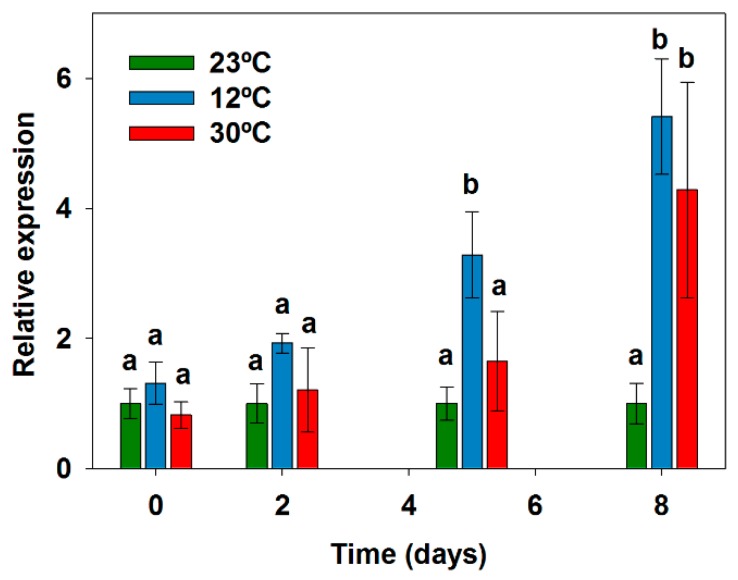
Expression of *sxtA* gene in *Aphanizomenon gracile* UAM529 under three different temperatures. Data represent relative expression of *sxtA* respective to 23 °C, which was set to 1 and placed on the left-hand side of each bar group to facilitate comparison. Letters indicate groups with significant differences (*p* < 0.05; one-way ANOVA and Holm-Sidak post-hoc test).

Temperature induced differences in the expression levels of *sxtA*, as can be observed in Figure 2. *SxtA* showed higher expression levels at the extremes of temperature (12 °C and 30 °C) than at the intermediate temperature of 23 °C throughout the 8-day growth period. Those differences were more evident (and statistically significant), especially on days 5 and 8 (Figure 2). Indeed, on day 8, relative expression values at the extremes of temperature were 4.3-fold (at 30 °C) and 5.4-fold (at 12 °C) higher than those at the reference temperature of 23 °C.

**Figure 3:**
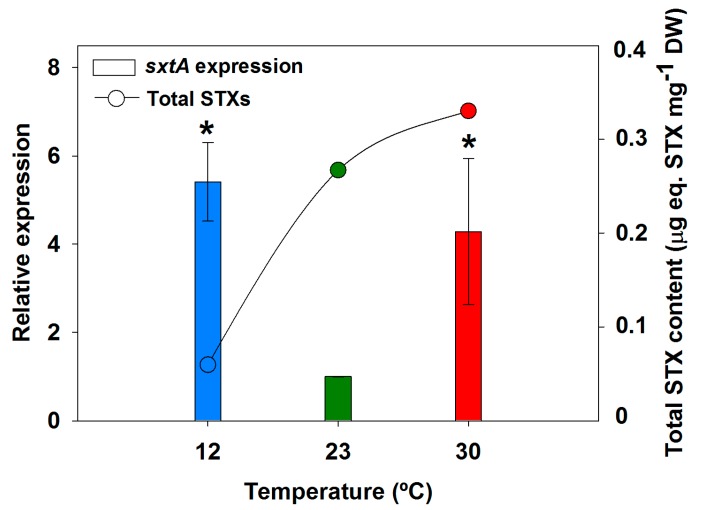
Relationship between total STX content and *sxtA* gene expression in *Aphanizomenon gracile* UAM529 after 8 days of growth. Vertical bars show relative expression values respective to 23 °C, which was set to 1 to facilitate comparison. Asterisks indicate significant differences of relative expression with 23 °C (*p* < 0.05; one-way ANOVA and Holm-Sidak post hoc test).

Assuming that *sxtA* is involved in the first step in the synthesis of STX, it could be expected that increased levels of *sxtA* expression would result in higher cellular contents of STX. In order to check this possible relationship, the highest expression levels of *sxtA* in our study (observed on day 8) were plotted against the STX contents on the same date (Figure 3). As expected, the clear overexpression of *sxtA* observed for 30 °C coincided with high STX content. In contrast, at 12 °C both parameters showed an inverse relationship, with the high *sxtA* expression levels coinciding with a very low STX content (Figure 3).

#### 2.3.2. Dynamics of STX-Transporter Genes *sxtM* and *sxtPer*

**Figure 4:**
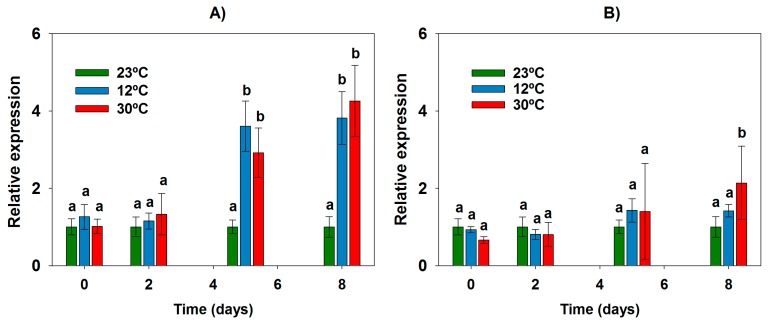
Expression of genes related to saxitoxin (STX) transport (*sxtM* and *sxtPer*) in *Aphanizomenon gracile* UAM529 under three different temperatures. Data represent relative expression of genes *sxtM* (**A**) and *sxtPer* (**B**) respective to 23 °C, which was set to 1 and placed on the left-hand side of each bar group to facilitate comparison. Letters indicate groups with significant differences (*p* < 0.05; one-way ANOVA and Holm-Sidak post hoc test).

The genes *sxtM* and *sxtPer*, encoding two transporters putatively involved in STX transport, were transcribed at the three temperatures studied throughout the entire 8-day growth period. As for *sxtA*, relative expression values of *sxtM* and *sxtPer* were calculated with 23 °C as the control condition showing the optimum physiological status as well as the lowest levels of extracellular STX. Temperature influenced *sxtM* expression (Figure 4A). Indeed, the *sxtM* expression levels at the extremes of temperature (12 °C and 30 °C) were higher than those of 23 °C throughout the 8 day-period, with differences being more marked (and statistically significant) on days 5 and especially on day 8 (3.8-fold overexpression at 12 °C; and 4.1-fold overexpression at 30 °C) (Figure 4A). Regarding *sxtPer*, the influence of temperature was less clear, with the three temperatures showing similar expression levels during the first 5 days of growth (Figure 4B). On day 8, there was an almost negligible overexpression at 12 °C (1.4-fold) and a slight (but statistically significant) overexpression at 30 °C (2.1-fold) compared to 23 °C (Figure 4B).

**Figure 5:**
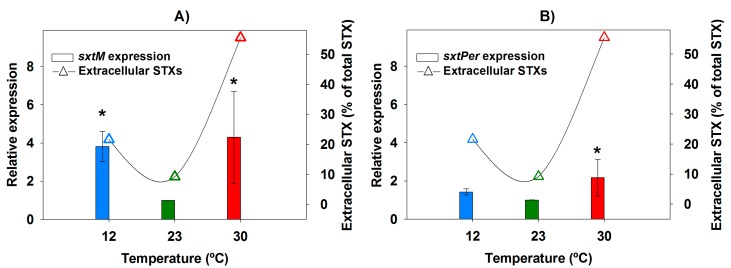
Relationship between extracellular STX share and expression of genes involved in STX transport (*sxtM* and *sxtPer*) in *Aphanizomenon gracile* UAM529 after 8 days of growth. Vertical bars show relative expression values of *sxtM* (**A**) and *sxtPer* (**B**) with respect to 23 °C, which was set to 1 to facilitate comparison. Asterisks indicate significant differences of relative expression with 23 °C (*p* < 0.05; one-way ANOVA and Holm-Sidak post hoc test).

In order to check whether the increased expression of the putative STX transporters *sxtM* and *sxtPer* was reflected into higher STX release outside the cells, the expression levels of the two genes were plotted against the extracellular STX share on day 8 as the date with the maximum differences in gene expression (Figure 5).

In the case of *sxtM*, the increased expression levels found at the extremes of temperature (12 and 30 °C) coincided with a larger extracellular STX share, especially at 30 °C (Figure 5A). Concerning *sxtPer* (Figure 5B), this relationship was unclear for both temperatures, and the large extracellular STX proportion at 30 °C (55.5%) coincided just with a slight overexpression of *sxtPer* (2.1-fold) compared to 23 °C.

### 2.4. Effects of Nitrogen Depletion at 23 °C 

Besides considering the effect of temperature in nitrogen-replete cultures shown so far, the possible effect of nitrate depletion from culture media was studied at the optimum temperature for growth (23 °C) (Table 3). Cultures growing in nitrate-depleted medium (BG11_0_), hence utilizing fixed atmospheric N_2_ as the sole nitrogen source, displayed a clearly reduced growth compared with those with nitrate (BG11 medium) (Table 3). This apparently worse physiological condition of BG11_0_ was also reflected into remarkably lower Chl *a* content (Table 3), as well as in a compromised membrane integrity, evidenced by higher PI fluorescence (Table S2 in Supplementary Material). Additionally, a decrease in DiBAC_4_(3) fluorescence indicated a certain hyperpolarisation of the membrane in BG11_0_ compared to BG11-grown cultures (Table S2 in Supplementary Material).

Regarding toxins, nitrate depletion at 23 °C did not induce differences in total STX production, whereas it notably affected the extracellular STX share (Table 3). As a matter of fact, cultures grown in BG11_0_ displayed an extracellular STX share 4.3-fold higher than those in BG11 (51.2% in BG11_0_ vs. 11.8% in BG11). Surprisingly, this high extracellular STX was not related at all to an enhanced transcription of *sxtM* and *sxtPer* since, together with *sxtA*, their expression levels in BG11_0_ were very similar to those of BG11-grown cultures (Table 3).

## 3. Discussion

Planktonic Nostocales cyanobacteria represent a hot topic among scientists because of their ability to produce almost all types of cyanotoxins known and their ecological plasticity linked to a presumable invasive behavior under global warming [20,21]. The present study provides insight into the ecophysiology of *Aph. gracile*—the most widely distributed STX producing-nostocalean so far [11]—adding to previous works in *Aph. gracile* strains from Spain [15] and Germany [22]. In synthesis, these studies evidenced that growth for *Aph. gracile* is optimum at water temperatures of 23–28 °C and afterwards decreases at high temperatures (28–30 °C), being outcompeted by co-occurring nostocalean with assumed subtropical-tropical origin (e.g., *Cyl. raciborskii*, *Sphaerospermopsis aphanizomenoides*, *Chrysosporum ovalisporum*) [22]. The high growth of *Aph. gracile* at low temperatures of 10–12 °C is remarkable, meaning it could have an early onset in spring, or even a prolonged autumn-winter growth. This apparently extended annual life cycle of *Aph. gracile* could pose an ecological advantage and warns water managers to monitor this species not just in summer but throughout the year, especially considering that global warming is predicted to even extend the length of vegetative growth periods in cyanobacteria in general [22].

Temperature influenced both STX production and *sxtA* transcription levels in *Aph. gracile* UAM529. The *sxtA*gene—encoding for a putative PKS catalyzing the first step in STX biosynthesis—is found in all STX-producing cyanobacteria so far, and as such it is the usual marker in phylogenetic and transcription analyses. Interestingly, we observed a temperature-dependent expression of *sxtA* gene with upregulation at extremes of temperature (12 °C and 30 °C) away from the optimum for growth (23 °C). However, *sxtA* expression and STX content did not show a direct relationship, as increased *sxtA* levels coincided with a clearly decreased STX content at 12 °C, and with a just slight toxin increase at 30 °C. Previous *sxtA* transcription works on factors other than temperature (Na^+^, pH, N sources) showed controversial results. The transcription of *sxtA*S*xtA* correlated with STX contents in cultures of *A. circinalis* or *Cyl. raciborskii* [18], while, in contrast, no relationship was found for the cyanobacterium *R. brooki* [19] or the dinoflagellate *Alexandrium minutum* [23].

These contradictory results on *sxtA* transcription vs. STX content might be explained by downstream regulation occurring at post-transcriptional or post-translational levels. Another possibility is a reduced activity of the enzyme SxtA under temperatures as low as 12 °C. For instance, the enzyme CyrA involved in the first biosynthetic step of another cyanotoxin (cylindrospermopsin, CYN) showed marked temperature-dependent patterns of activity [24,25]. Furthermore, it has to be noted that STX biosynthesis requires not just *sxtA* but the combined action of at least 14 genes of the *sxt* cluster [12], which could be suffering differential regulation by temperature (e.g., at 12 °C *sxtA* might be upregulated while other downstream *sxt* genes may be downregulated). In this sense, future works should try to analyze the simultaneous response of several sxt-biosynthesis genes either by quantitative PCR or by more potent (yet qualitative) tools such as RNA-Seq.

Temperatures outside the optimum for growth induced increased extracellular STX share in *Aph. gracile*, relevant at 12 °C (16% of extracellular STXs) and especially remarkable at 30 °C (54%). This supports previous observations of high extracellular STXs (7%–35% of total STXs) in *Aph. gracile* and *C. issatschenkoii* [15,16] and also opens the possibility of shares over 50% at high temperatures of 30 °C. Although 30 °C is an unlikely water temperature in deep stratified lakes where *Aph. gracile* thrives, temperatures of 28–30 °C can be reached in Mediterranean waterbodies during summers under certain conditions such as in very shallow urban ponds (e.g., Juan Carlos I pond in the city of Madrid, see [22]) or in lakes/reservoirs receiving cooling waters of thermal or nuclear power plants [26].

Interestingly, STXs and CYN, despite being two distant groups of cyanotoxins, both show a high extracellular toxin proportion [11]. This may be somewhat related to them sharing transporters of the same family (NorM-MATE), namely *cyrK* for CYN and *sxtM* and *sxtF* for STX. Contrastingly, another major group of cyanotoxins, microcystins, is characterized by low extracellular proportions during exponential growth (below 10%) and instead it shows a putative transporter of a different family (ABC) [27].

STX export outside the cyanobacterial cell via putative NorM-MATE-type transporters is still not fully understood. Current knowledge is restricted to the elegant *in-silico* protein-structure analyses by Soto-Liebe et al. [14] and transcription studies of *sxtM* and *sxtF* in *A. circinalis, Cyli. raciborskii, and R. brokii* subjected to factors other than temperature (pH, Na^+^, and N sources) (Table 4, and references therein). The present study contributes the novel finding of a temperature-dependent regulation of *sxtM* in *Aph. gracile*, with an upregulation at extremes of temperature (12 and 30 °C) outside the optimum for growth (23 °C). Temperature-dependent regulation of NorM-MATE proteins has been already observed in other Gram-negative bacteria such as the pathogens *Erwinia amylovora* [28] and *Yersinia* [29]. Importantly, in *Aph. Gracile,* the higher expression levels of *sxtM* (3.8-fold at 12 °C and 4.1-fold at 30 °C) coincided with increased extracellular STX share, especially at 30 °C, suggesting an increase in active STX transport because of greater amounts of available SxtM protein. Additionally, cultures at 30 °C showed hyperpolarized cell membrane compared to the reference temperature of 23 °C (Supplementary Table S1), which could have somewhat influenced ion transport (e.g., Na^+^) through the membrane and indirectly affected STX export. Despite the interest of this finding, deeper studies on cell membrane are needed to understand whether this is a reversible phenomenon as well as its actual effects on STX transport.

Besides NorM-MATE protein SxtM, STX export might also occur in *Aph. gracile* via SxtPer, a second transporter phylogenetically distant from SxtM whose presence is only confirmed in 3 STX-producing cyanobacteria [14,30] and very recently in one dinoflagellate [31]. According to sequence homologies, SxtPer protein is classified as a permease of the drug/metabolite transporter Superfamily (DMT) fitting within the TMS Drug/Metabolite Exporter (DME) Family involved in metabolite and drug extrusion [14]. As far as we know, the present study shows the first results on the regulation of *sxtPer* transcription under environmental gradients. Yet, *sxtPer* expression was slightly upregulated at 30 °C (2.1-fold) compared to 12 and 23 °C, and *sxtPer* expression could not be unequivocally correlated either with temperature or with the proportion of extracellular STX, unlike in *sxtM*. This raises many open questions regarding the actual implication of *sxtPer* in STXs transport. A possible hypothesis is that *sxPer’s* main role is the export of metabolites or antibiotic/drugs with a marginal transport of only some specific STX analogues [32]. This may be addressed by future works on the topic, e.g., those similar to the kinetic studies performed by Soto-Liebe in *sxtM*, to determine the hypothetical affinity of *sxtPer* protein for the different STX analogues.

Apart from temperature, nitrate depletion has also induced increased STX production in *Aph. gracile* (this study; and [15]), *A. circinalis, R. brookii,* and *C. issatschenkoi* [16,33,34], and also in extracellular release in *Aph. gracile* and *C. issatschenkoii* (this study; and [15,16]). In our study, the extracellular share in nitrate-depleted *Aph. gracile* was even higher (51%) than that found by Casero et al. (35%) [15]. However, our results are likely influenced by damages in the cell membrane (see Supplementary Table S2), as well as by cell lysis due to growth decay, especially after day 5, which may not have happened in the 2-day growth experiments by Casero et al. [15]. In any case, the influence of nitrogen on STX synthesis and/or transport is reasonable as binding boxes of NtcA, the master regulator of N metabolism, have been found within *sxt* cluster as potential regulators of STX synthesis [19]. In spite of these hints, a hypothetical transcriptional influence of nitrogen remains elusive as neither our study in *Aph. gracile* nor that by Stucken et al. in *R. brookii* [19] could find an influence of nitrogen sources on *sxt* gene expression so far. In this context, a very interesting proteomic study in STX-producing *A. circinalis* opened the gate to post-translational regulation [32]. The authors hypothesized that STX content may increase under high C:N ratio (which could be occurring with low nitrogen media, e.g., in BG11_0_ medium) via the regulation of SxtS protein by 2-oxoglutarate [32]. Keeping all of this in mind, further research efforts may focus not just on transcription but also on proteomics, with a focus on disentangling the influence of C:N ratios, alone or in combination with other factors that remain unstudied in *Aph. gracile* which have influenced *sxtM* expression such as Na^+^ and pH (see Table 4).

## 4. Conclusions

In conclusion, this work evidences a temperature-dependent STX production and release in *Aph. gracile*. Our ecophysiology data indicate that the monitoring of *Aph. gracile* for water management purposes should cover not just summer but also spring and autumn at water temperatures of 10–23 °C. Extrapolating our results to blooms dominated by STX-producing *Aph. gracile*, the maximum risk for high dissolved STX concentrations might occur (even without bloom decay) during very hot periods reaching water temperatures of 28–30 °C, and especially under low nitrate concentrations in water. Regarding the molecular mechanisms involved, the temperature-dependent STX synthesis observed may be partially explained by a transcriptional regulation of *sxtA* at high temperatures. The influence of temperature on STX export, on the other hand, might be mainly due to transcriptional upregulation of *sxtM* at both low and high temperatures outside the optimum. Finally, this work opens interesting avenues regarding, among others: the role of *sxtPer* gene; a possible post-transcriptional/post-translational influence of nitrogen on *sxt* regulation; and effects of cell membrane hyperpolarization on STX transport. Those challenges might be addressed by future studies involving advanced –omics with multi-gene analyses to fully understand the complex regulation of STX synthesis and transport in cyanobacteria while providing clues on the still unsolved role of saxitoxins.

## 5. Materials and Methods

### 5.1. Culture Conditions

Temperature experiments were performed in cultures of the cyanobacterial strain *Aph. gracile* UAM529 from Rosarito reservoir (Tiétar river, Toledo, Central Spain), isolated in a previous study by Cirés and co-workers [21] and maintained at Autónoma de Madrid University culture collection (UAM, Madrid, Spain).

Aliquots of *Aph. gracile* UAM529 were cultured at three different temperatures (12 °C, 23 °C, and 30 °C) under a batch regime for 8 days. Temperatures were selected to cover the range for vegetative growth of *Aph. gracile* in freshwater bodies of temperate latitudes [2,3]. Cultures were pre-acclimated to each of the temperatures during three generation times, after which each experiment was started at an O.D. _750 nm_ of 0.2 corresponding to a biomass of approximately 0.1 g DW L^−1^. Cultures were grown in triplicate in Erlenmeyer flasks containing 500 mL of BG11 culture medium [4] under white light illumination (30 µmol photons m^−2^ s^−1^), simulating 16:8h light:dark photo-period and supplemented with sterile air bubbling.

In order to test the possible influence of the nitrate depletion in the culture media, one additional experiment was set at 23 °C, including cultures of *Aph. gracile* UAM529 grown in BG11_0_ medium [4]—not containing a source of combined nitrogen—under the same light and bubbling conditions detailed above for the temperature experiments.

### 5.2. Growth Dynamics 

The growth of *Aph. gracile* UAM529 was monitored throughout the 8 day period by determining the dry weight (g L^−1^) and the chlorophyll *a* concentration (µg L^−1^) in samples taken at four different time points (days 0, 2, 5, and 8).

The dry weight (DW) in the light-intensity experiments was determined from pre-desiccated glass microfiber filters GF/F (Whatman, Maidstone, UK), saturated with culture material (10 mL) after low-vacuum filtration. The filters were desiccated at 65 °C and periodically weighed until reaching a constant weight (typically in 24 h). Dry weight (g) was relativized to the filtered volume and expressed as g DW L^−1^.

The chlorophyll *a* (Chl *a*) concentration was determined from biomass-saturated GF/F filters (containing 10 mL of culture material) that were kept at −20 °C until analysis. The filters were extracted twice into 90% (*v*/*v*) acetone. Filters were sonicated on an ultrasonic bath 2510 (Branson Ultrasonics, Saint Louis, MO, USA) for 5 min and extracted at 4 °C for 12 h. The two extracts were centrifuged (1385× *g*, 10 min) and pooled together for the spectrophotometric determination. The O.D._750nm_ and O.D._665nm_ of the extract were measured by a UV-Vis Hitachi U-2000 spectrophotometer (Shimadzu, Kyoto, Japan) and converted into Chl *a* concentrations (µg L^−1^) according to [35].

### 5.3. Alterations in Membrane Integrity and Membrane Potential by Flow Cytometry

Potential alterations in membrane properties of *Aph. gracile* UAM529 after 8 days of growth were tracked by fluorescence labelling of cells coupled with flow cytometry [36,37] in 10 mL culture samples taken on day 8 of each of the temperature experiments. Alterations in membrane permeability were assessed by propidium iodide (PI) permeability bioassay. Alterations in membrane potential were studied using DiBAC_4_(3) fluorescence dye. PI (538 nm/617 nm) (Life Technologies, Carlsbad, CA, USA) is non-permeable for healthy cell membranes, while it can penetrate cells with damaged or disrupted cell membranes. DiBAC_4_(3) [DiBAC_4_(3) (Bis-(1,3-Dibutylbarbituric Acid)Trimethine Oxonol)] (490 nm/516 nm) (Life Technologies, Carlsbad, CA, USA) can enter depolarized cells where it binds to intracellular proteins or membranes. Increased depolarization of cell membranes results in additional influx of the anionic dye and an increase in fluorescence. Conversely, hyperpolarization is indicated by a decrease in fluorescence. Stock solutions of PI (1 mg mL^−1^) and DiBAC_4_(3) (0.5 mg mL^−1^) were prepared in water (PI) and dimethyl sulfoxide (DiBAC_4_(3)) (DMSO 100%, Sigma-Aldrich, Saint Louis, MO, USA) in dim light conditions to avoid degradation, and were kept frozen (−20 °C until use). For cell staining, aliquots of cyanobacteria cells were incubated with 2.5 µg mL^−1^ of PI and 0.5 µg mL^−1^ of DiBAC_4_(3) (final concentrations) for 10 min. Changes in fluorescence were analysed by flow cytometry in a Cytomix FL500 MPL flow cytometer equipped with an argon-ion excitation wavelength (488 nm), forward (FS), and side (SS) light scatter detectors, and four fluorescence detectors (FL1:525 nm, FL2:575 nm, FL3:620 nm, and FL4:675 nm) (±20 nm) (Beckman Coulter Inc., Fullerton, CA, USA). Operating conditions were adjusted as described in [36,38]. At least 10,000 events were counted per acquisition. Chlorophyll red autofluorescence was collected in FL4; PI was acquired at 617 nm in FL3 and DiBAC_4_(3) was acquired at 516 nm in FL1.

### 5.4. Analysis of the Expression of Genes sxtA, sxtM, and sxtPer by qPCR

The expression of one gene putatively involved in the first step of STXs biosynthesis (*sxt*A) and two genes putatively involved in STXs transport (*sxtM* and *sxtPer*) was analyzed by Real Time qPCR in samples of *Aph. gracile* UAM529 taken at four different time points (days 0, 2, 5, and 8) from each of the temperature experiments.

#### 5.4.1. Total RNA Extraction and cDNA Synthesis

Total RNA was extracted from 10 mL culture samples that were centrifuged (4300× *g,* 5 min) at the same temperatures of each of the experiments (12 °C, 23°C, and 30 °C, respectively). The supernatants were discarded and 1 mL of RNA Later (Ambion, Waltham, MA, USA) was added to the cell pellets, which were incubated at 4 °C overnight and subsequently stored at −80 °C until RNA extraction. The extraction and purification of total RNA was carried out through the RNeasy Mini Kit (QIAGEN, Hilden, Germany). Culture samples were centrifuged (4300× *g*, 5 min) to remove RNA Later, and the pellets were incubated at room temperature with 1 mL of QIAzol Lysis Reagent (Qiagen, Hilden, Germany) for 5 min. Afterwards, the entire content was transferred to Eppendorf tubes and lysed by vigorous shaking in a Tissue Lyser (Qiagen, Hilden, Germany) at 30 Hz for 5 min using 5 mm stainless-steel beads. The lysate was treated with 200 µL of chloroform, vortexed for 5 min, incubated at room temperature for 2 min, and subsequently centrifuged at 4 °C (12,000× *g*, 15 min). The aqueous phase was separated and subjected to extraction using the RNeasy Mini Kit (QIAGEN, Hilden, Germany), following the manufacturer instructions from this step onwards. In order to minimize interferences by genomic DNA, during RNA extraction samples were incubated with the RNase-Free DNase (QIAGEN, Hilden, Germany) at room temperature for 15 min. After extraction, total RNA quantification was performed by spectrophotometry in a Nanodrop ND-1000 UV spectrophotometer. RNA integrity and quality was checked using an Agilent Bioanalyzer 2100 (Agilent Technologies, Santa Clara, CA, USA).

Synthesis of cDNA was carried out by subjecting total RNA samples to Reverse-Transcription through the High-Capacity RNA-to-cDNA™ Kit (Applied Biosystems, Waltham, MA, USA), following the manufacturer recommendations. For such purpose, 250 ng of RNA was brought to PCR tubes and mixed with 0.5 µL of 20X inverse transcriptase, 5 µL of 2X reaction buffer, and nuclease-free water to make a total volume of 10 µL. PCR tubes were incubated in a thermocycler (37 °C, 60 min.; 95 °C, 5 min) and resulting cDNA samples were preserved at −20 °C until analyzed by Real Time qPCR.

#### 5.4.2. Real Time qPCR

Specific primers for the amplification of genes 16S rRNA, *sxtM*, and *sxtPer* were designed using Primer Express 3.0 software (Applied Biosystems, Darmstadt, Germany) (Table 5) from sequences of *Aphanizomenon gracile* UAM529 available at NCBI Genbank [39] for 16S rRNA (accession number JN886009) and those available at European Nucleotide Archive [40] for *sxtM* and *sxtPer* (accession number LT549447 for the *sxt* cluster of *Aph. gracile* UAM529). Primers for *sxtA* were obtained from [18] (Table 4). The specificity and homogeneity of amplicons was confirmed by sequencing PCR products in an ABI Prism 3730 Genetic Analyzer (Applied Biosystems, Waltham, MA, USA). The efficiency of amplification for each primer pair (Table 5) was determined by linear regression analyses from cDNA standard curves.

Real Time qPCR was performed in 10 µL volume, including 5 µL of Power SYBR Green PCR Master Mix (Applied Biosystems, Waltham, MA, USA) and 0.25 µL of each primer (250 nM). Amplification reactions were carried out in a AB7900HT Fast Real Time cycler (Applied Biosystems, Waltham, MA, USA) under conditions as follows: one cycle at 50 °C for 2 min and one cycle at 95 °C for 10 min, followed by 40 cycles of 95 °C for 15 s and 60 °C for 60 s. Each reaction was run in triplicate. Two types of negative controls were included for each gene in every run: a no template control, and a negative control of total RNA as template to verify the absence of genomic DNA in the sample.

Gene expression data from the qPCR amplification were evaluated using the threshold cycle (Ct) values. The 16S rRNA gene was used as a control to normalize the expression levels of target genes. Relative transcription was determined using the 2^−ΔΔCt^ method [41] with 23 °C as control condition, where ΔΔCt = (Ct_target_ − Ct_16S_) _temperature x_ − (Ct_target_ − Ct_16S_) _23 °C_, following the recommendations by the Fast Real Time Cycler manufacturer [42].

### 5.5. Quantification of Intracellular and Extracellular Saxitoxins

Culture samples for the determination of intracellular and extracellular STXs were taken at for different time points (days 0, 2, 5, and 8) throughout the experimental period. Intracellular STX was determined from GF/F filters (Whatman, Maidstone, UK) saturated with 10 mL of culture material after gentle-vacuum filtration. The filtrate was collected for the determination of extracellular STX. The filters and filtrates were kept at −20 °C until extracted and analysed using the Abraxis Saxitoxin enzyme-linked immunosorbent assay (ELISA) (Abraxis LLC, Warminster, PA, USA). According to the kit manufacturer, the ELISA antibody targets primarily STX (100% cross reactivity) and shows a slight interaction with 2 variants—decarbamoylsaxitoxin (29% cross reactivity) and neosaxitoxin (1.3%)—detected in low amounts in *Aph. gracile* UAM529 [21].

STX retained in filters was extracted twice into methanol 80% (*v*/*v*), as recommended by the ELISA kit manufacturer. In each extraction step, filters were sonicated on a ultrasonic bath 2510 (Branson Ultrasonics, Saint Louis, MO, USA) for 10 min, and extracted at 4 °C for 1 h. The two extracts were centrifuged (4300× *g*, 15 min) and pooled together for the determination of intracellular STX. The extracellular STX in the filtrate was directly analysed by ELISA. Both the intracellular extracts and the extracellular samples were centrifuged (10,000× *g*, 5 min), and the supernatants were diluted 150–1000 fold (intracellular extracts) and 140–250 fold (extracellular samples) with 1X dilution buffer provided with the ELISA kit in order to fit concentrations within the quantitative range of the kit (0.02–0.4 µg equiv. STX L^−1^). All standards and samples were run in duplicate following the manufacturer instructions. ELISA absorbance readings were performed at 450 nm on a Synergy HT multi-mode microplate reader (BioTek, Wilusky, VT, USA). STX concentrations (µg equiv. STX L^−1^) were standardized to dry weight in order to obtain saxitoxin contents (µg equiv. STX mg^−1^ DW). Total saxitoxin contents were calculated as the sum of the intracellular and the extracellular contents of each culture sample.

### 5.6. Statistical Analysis

All groups of data were analyzed for normality by the Shapiro–Wilk test and for homoscedasticity by the Levene test to ensure that the assumptions of parametric tests were met. Pairwise comparisons were performed using the *t* test. Multiple comparisons were carried out using the one-way ANOVA test. Post hoc comparisons were carried out using the Holm-Sidak test. A significance level of *p* = 0.05 was established for all the tests. Statistics were performed with the Sigmaplot software (version 11.0, 2008, Systat Software, San Jose, CA, USA).

## Figures and Tables

**Table 1:** Growth parameters of *Aphanizomenon gracile* UAM529 under three different temperatures. Data show mean ± standard deviation of three replicates (n = 3). Superscript letters refer to groups with statistically significant differences (*p* < 0.05; one-way ANOVA and Holm-Sidak post hoc test).

Temperature (°C)	Growth Rate (day^−1^)	Chl *a* Content (mg g^−1^ DW)
12	0.10 ± 0.02 ^a^	8.6 ± 1.6 ^a^
23	0.17 ± 0.03 ^b^	13.6 ± 2.4 ^b^
30	0.07 ± 0.01 ^a^	9.9 ± 3.1 ^a^

Chl *a* = chlorophyll *a*; DW= dry weight.

**Table 2:** Average saxitoxin contents in *Aphanizomenon gracile* UAM529 under three different temperatures. Data show average values over the entire 8-day experimental period ± standard deviation (*n* = 12, corresponding to 4 time points and 3 biological replicates on each time point). Superscript letters refer to groups with statistically significant differences (*p* < 0.05; one-way ANOVA and Holm-Sidak post hoc test).

Temperature (°C)	Total STX Content (µg mg^−1^ DW)	Total STX Content (µg mg^−1^ Chl *a*)	Extracellular STX (% of Total STX)
12	0.08 ± 0.03 ^a^	8.7 ± 2.2 ^a^	16.9 ± 10.2 ^a^
23	0.20 ± 0.08 ^b^	14.0 ± 4.3 ^a^	11.8 ± 6.1 ^a^
30	0.25 ± 0.07 ^b^	28.6 ± 13.7 ^b^	53.8 ± 15.5 ^b^

Chl *a* = chlorophyll *a*; DW = dry weight: STX = saxitoxin.

**Table 3:** Effect of nitrate depletion in *Aphanizomenon gracile* UAM529 grown at 23 °C in two different culture media (BG11, with nitrate, and BG11_0_, without nitrate). Data show mean ± standard deviation of three replicates (*n* = 3). Asterisks indicate significant differences of BG11_0_—grown culture compared to BG11 (*p* < 0.05, *t*-test). Expression data of BG11_0_ are relative to BG11 expression, which was set to 1 to facilitate comparison.

Culture Medium	Ecophysiology		Toxin Production/Release		Relative Expression		
	Growth Rate (day^−1^)	Chl *a* Content (mg g^−1^ DW)	Total STX Content (µg mg^−1^ DW)	Extracellular STX (%)	*sxtA*	*sxtM*	*sxtPer*
BG11	0.17 ± 0.03	13.6 ± 2.4	0.20 ± 0.08	11.8 ± 6.1	1.0 ± 0.01	1.0 ± 0.02	1.0 ± 0.01
BG11_0_	0.05 ± 0.01 *	7.6 ± 1.9 *	0.15 ± 0.04	51.2 ± 20.2 *	1.5 ± 0.5	1.2 ± 0.5	1.1 ± 0.3

Chl *a* = chlorophyll *a*; DW = dry weight: STX = saxitoxin.

**Table 4:** Summary of studies on environmental regulation of saxitoxins transporters expression in different cyanobacterial species.

Organism	Factor	Effect on Extracellular STX Release	Effect on the Expression of STX Transporters		Reference
			*sxtM*	*sxtPer*	
*Aphanizomenon* *gracile*	Temperature (12°C and 30°C vs. 23 °C)	4-fold higher at 30 °C than at 23 °C	Upregulation (2.9–4.3X) at 30 °C Upregulation (3.6–3.8X) at 12 °C	Upregulation (2.1X) at 30 °C	This study
	Nitrate (absence—BG11_0_- vs. presence—BG11-)	4-fold higher without nitrate (BG11_0_)	NS	NS	This study
*Anabaena* *circinalis*	Ph (9 vs. 7)	Higher at pH 9 (exact value not provided)	Downregulation at pH 9 (59X) vs. pH 7	NP	[18]
	Na^+^ (10 mM vs. 1.3 mM ^1^)	Higher at 10 mM (exact value not provided)	Downregulation at 10 mM (2.7X) vs. 1.3 mM	NP	[18]
*Cylindrospermopsis* *raciborskii*	pH (9 vs. 7)	Higher at pH 9 (exact value not provided)	Upregulation at pH = 9 (24X) vs. pH 7	NP	[18]
	Na^+^ (10 mM vs. 1.3 mM ^1^)	Higher at 10 mM (exact value not provided)	Upregulation at 10 mM (2.7X) vs. 1.3 mM	NP	[18]

^1^ 1.3 mM is the sodium concentration in Jaworski’s medium used as control. NS, differences not statistically significant; NP, gene not present in the cited species.

**Table 5:** qPCR primer sequences, efficiencies, and amplicon sizes.

Gene	Primer	Sequence (5’–3’)	Amplicon Size (bp)	Slope; R^2^	Efficiency (%) ^1^	Source
16S rRNA	q16grF q16grR	GAGAGACTGCCGGTGACAAA TGCCCTTTGTCCGTAGCATT	106	−3.24 0.995	103	This study
*sxtA*	jrtPKSF jrtPKSR	GGAGTGGATTTCAACACCAGAA GTTTCCCAGACTCGTTTCAGG	147	−3.38 0.999	98	[18]
*sxtM*	qMgrF qMgrR	GAAGCACGAGTCAGCCTACA CAAAGCACCACCAGCCAAAA	129	−3.29 0.998	101	This study
*sxtPer*	qPERgrF qPERgrR	CTGGGCGAGACATTTGAGA GCACAGAGACAGGCGAACTA	116	−3.37 0.993	98	This study

^1^ Efficiencies calculated as E = (10^−1/slope^ − 1) × 100.

## Supplementary Materials

The following are available online at www.mdpi.com/2072-6651/9/10/322/s1, Figure S1: Growth curves of *Aphanizomenon gracile* UAM529 under three different temperatures. Error bars indicate standard deviation of three replicates (*n* = 3), Table S1: Flow cytometry analysis of membrane integrity and membrane potential in *Aphanizomenon gracile* UAM529 under three different temperatures, Table S2: Flow cytometry analysis of membrane integrity and membrane potential in *Aphanizomenon gracile* UAM529 under two different culture media with combined nitrogen (BG11) and without combined nitrogen (BG11_0_).
