# Supplementary material for: Temperature Influences the Production and Transport of Saxitoxin and the Expression of sxt Genes in the Cyanobacterium Aphanizomenon gracile
Source: Toxins (Basel). 2017 Oct 13;9(10):322. doi: 10.3390/toxins9100322 (PMC5666369; doi:10.3390/toxins9100322)
Supplement: Supplementary file 1 [file toxins-09-00322-s001.pdf]

Supplementary Materials: Temperature Influences the Production and Transport of Saxitoxin and the Expression of *sxt* Genes in the Cyanobacterium *Aphanizomenon gracile*

Samuel Cirés, Adrián Delgado, Miguel González-Pleiter and Antonio Quesada

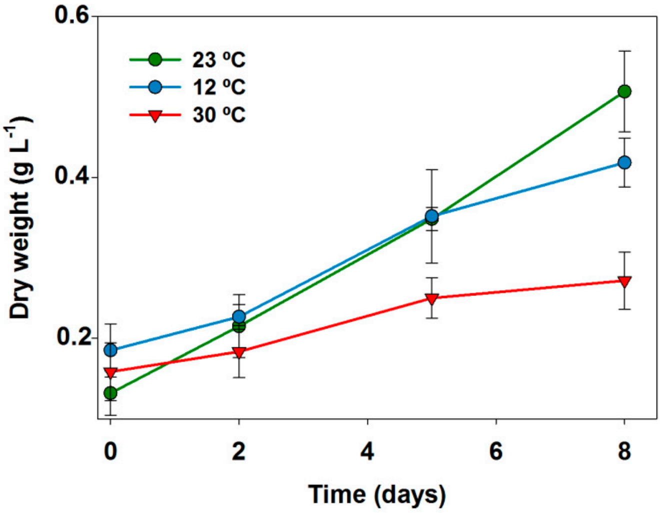

Figure S1. Growth curves of *Aphanizomenon gracile* UAM529 under three different temperatures. Error bars indicate standard deviation of three replicates (n = 3).

Table S1. Flow cytometry analysis of membrane integrity and membrane potential in *Aphanizomenon gracile* UAM529 under three different temperatures. Results are expressed as relative fluorescence units (average ± standard deviation) setting 23 °C as 100% to facilitate comparison. Asterisks indicate significant differences respective to 23 °C ($p < 0.05$; one-way ANOVA). PI (propidium iodide) and DiBAC4(3) ($DiBAC_4(3)$ (Bis-(1,3-Dibutylbarbituric Acid)Trimethine Oxonol) are fluorescent dyes for membrane permeability and membrane potential, respectively.

**Temperature**	**Average value of medians (*n* = 6)**	
**(°C)**	**PI**	**DiBAC4(3)**
23	100.0 ± 0.4	100.0 ± 16.6
12	101.2 ± 0.1	89.1 ± 12.0
30	100.2 ± 1.7	12.6 ± 2.7*

Table S2. Flow cytometry analysis of membrane integrity and membrane potential in *Aphanizomenon gracile* UAM529 under two different culture media with combined nitrogen (BG11) and without combined nitrogen ($BG11_0$). Results are expressed as relative fluorescence units (average ± standard deviation) setting BG11 as 100% to facilitate comparison. Asterisks indicate significant differences respective to BG11 ($p < 0.05$; t test). PI (propidium iodide) and $DiBAC_4(3)$ (Bis-(1,3-Dibutylbarbituric Acid)Trimethine Oxonol) are fluorescent dyes for membrane permeability and membrane potential, respectively.

**Culture**	**Average value of medians (*n* = 6)**	
**medium**	**PI**	**DiBAC4(3)**
BG11	100.0 ± 16.6	100.0 ± 0.4
$BG11_0$	95.5 ± 2.2*	74.2 ± 5.1*
